# Oxidative Stress in Disease and Aging: Mechanisms and Therapies

**DOI:** 10.1155/2016/8786564

**Published:** 2015-12-10

**Authors:** Claudio Cabello-Verrugio, Marta Ruiz-Ortega, Matias Mosqueira, Felipe Simon

**Affiliations:** ^1^Laboratory of Biology and Molecular Physiopathology, Department of Biological Sciences, Faculty of Biological Sciences and Faculty of Medicine, Universidad Andres Bello, Santiago, Chile; ^2^Millennium Institute on Immunology and Immunotherapy, Santiago, Chile; ^3^Cellular Biology in Renal Diseases Laboratory, IIS-Fundación Jiménez Díaz, School of Medicine, Universidad Autónoma Madrid, Madrid, Spain; ^4^Medical Biophysics Unit, Institute of Physiology and Pathophysiology, Heidelberg University, Im Neuenheimer Feld 324, R414, Heidelberg, Germany; ^5^Laboratory of Integrative Physiopathology, Department of Biological Sciences, Faculty of Biological Sciences and Faculty of Medicine, Universidad Andres Bello, Santiago, Chile

There is an increase in the population affected by chronic disease and aging during the last decades in which the oxidative stress is common factor in its development. Cellular oxidative stress is defined as an imbalance between the formation of reactive oxygen species (ROS) and antioxidant defense mechanisms. Due to the broad and profound biological effects of ROS, in the last years numerous experimental and clinical studies have focused their attention on the participation of oxidative stress as a key regulator in chronic pathological status and aging.

This special issue of OSDA is devoted to the new and relevant findings about the mechanisms by which the altered balance between ROS and cellular antioxidant machinery induce oxidative damage during chronic disease or aging. Among other manuscripts in this special issue that are equally recommended by the editors, it is interesting to comment on the following manuscripts.

M. A. Gómez-Marcos et al. tested the hypothesis whether superoxide dismutase (SOD) serum levels are correlated with vascular structure and function in hypertensive and type 2 diabetic patients. They showed negative correlations between SOD and pressure wave velocity, peripheral and central augmentation index, ambulatory arterial stiffness index, pulse pressure, and plasma HDL-cholesterol and positive correlations between SOD and plasma uric and triglycerides, suggesting that SOD serum levels turn in a marker for cardiovascular alterations in hypertensive and diabetic patients.

Y. Ting Lam discusses the roles of ROS in regulating stem and progenitor cell function, highlighting the impact of unbalanced ROS levels on endothelial progenitor cells dysfunction and the association with age-related impairment in ischemia-induced neovascularization. Furthermore, it discusses strategies that modulate the oxidative levels of stem and progenitor cells to enhance the therapeutic potential for elderly patients with cardiovascular disease.

O. Kost et al. examine a nanoscale therapeutic modality for the eye on the base of antioxidant enzyme SOD1, termed “nanozyme.” They show that the nanozyme was much more effective compared to the free enzyme in decreasing uveitis manifestations, considering SOD1-containing nanozyme in a potentially useful therapeutic agent for the treatment of ocular inflammatory disorders.

H. Liu et al. tested the antioxidant and anti-inflammatory effects of polydatin, an active compound isolated from* Polygonum cuspidatum* Sieb. et Zucc. roots on renal ischemia reperfusion model. The authors reported that polydatin significantly improved renal function after dose dependent increased expression of Akt phosphorylation and strong suppression of tumor necrosis factor-*α*, interleukin-1*β*, cyclooxygenase-2, inducible nitric oxide (NO) synthase and NO levels, and prostaglandin E-2. These results together open a strategy for the prevention and treatment of acute renal ischemia and reperfusion.

Y. Yang and S. Li investigated the protective roles of dandelion extracts used in the traditional Chinese and traditional medicine in the treatment of skin diseases. They found that dandelion extracts, especially leaf and flower extracts, are potent protective agents against UVB damage and H_2_O_2_-induced cellular senescence in HDFs by suppressing ROS generation and metalloproteinase activities and improving UVB absorption.

These manuscripts mentioned together with others contained in this special edition show that diverse antioxidant strategies have significant improvement on the pathological status reported. We hope that the readers of this special issue appreciate the progress and the new strategies developed in the field oxidative stress associated with disease and aging.

